# Exploring the effectiveness of physical health check interventions for people with severe mental illness: a systematic review of qualitative and quantitative evidence

**DOI:** 10.1186/s12913-026-14305-8

**Published:** 2026-03-21

**Authors:** Rachel L. Hawkins, Joao Craveiro, Ian Kellar, Jennifer V. E. Brown

**Affiliations:** 1 Sheffield Centre for Health and Related Research, University of Sheffield, Sheffield, UK; 2 School of Psychology, University of Sheffield, Sheffield, UK; 3 Department of Health Sciences, University of York, York, UK

**Keywords:** Severe mental illness, Physical health checks, Systematic review

## Abstract

**Background:**

People living with severe mental illness (SMI) have a life expectancy up to twenty years shorter than the general population. Among the causes for this health inequity is the increased risk of cardiovascular and metabolic conditions. Physical health checks were introduced to proactively care for the physical health of people with SMI. However, uptake to health checks in the United Kingdom are suboptimal and health inequities remain. The effectiveness of interventions on outcomes of physical health checks has yet to be properly examined, along with what supports or hinders their implementation. This review aimed to assess the effectiveness of health check interventions for changing physical health markers in the SMI population.

**Methods:**

Studies examining the impact of outpatient and community interventions on cardiovascular and metabolic health markers of adults with SMI were collated. Five databases were searched (Web of Science, CENTRAL, PsychInfo, SCOPUS, Medline). The Mixed Methods Appraisal Tool critically appraised studies and analysis followed the Joanna Briggs Institute Convergent Segregated approach to Mixed-Methods Systematic Reviews.

**Results:**

Eight articles (7 quantitative, 1 qualitative), totalling 1,828 participants, were included. Five found significantly positive effects on primary outcome(s). Successful studies improved health checks by integrating an extra layer of care (i.e. additional multidisciplinary teams) to existing services, whereas unsuccessful ones looked to improve checks within existing services. However, no marker was significantly improved across a majority of interventions. Four studies addressed barriers/facilitators to implementation success, revealing five themes.

**Conclusions:**

Current evidence is limited for understanding effective interventions on physical health checks, due to inconsistent core outcomes and reporting of interventions. Knowledge of implementation is significantly limited. The difference in outcomes across implementation strategies suggests a focus on equity over quality might be beneficial in the short-term. No studies embedded co-production within the development of physical health check interventions, highlighting a priority area for future research.

**Trial registration:**

https://doi.org/10.17605/OSF.IO/UWC2M.

**Supplementary Information:**

The online version contains supplementary material available at 10.1186/s12913-026-14305-8.

## Introduction

People living with severe mental illness (SMI), conditions such as schizophrenia, bipolar disorder, severe depression, schizoaffective disorder, and psychosis, face significant health inequalities, resulting in a life expectancy approximately 15 to 20 years shorter than the general population [1]. This early mortality is largely due to chronic physical health conditions such as cardiovascular disease (CVD), respiratory disease, liver disease, diabetes, and some cancers [2]. Evidence shows that these conditions occur at much higher rates (1.4–2.0 times higher) in people with SMI compared with the general population [3, 4].

Several factors contribute to this increased risk: people with SMI often experience higher rates of smoking, poor nutrition, and limited physical exercise, leading to cardiometabolic risks at a younger age [5]. Antipsychotic medications, commonly used to manage SMI, also contribute to weight gain, glucose intolerance, and poor cardiovascular (CV) health [6]. Furthermore, lack of access to community services, accessible information, and preventative interventions for people with SMI is also a known issue [5, 7]. In addition, physical illnesses in this population go underdiagnosed or undertreated due to “diagnostic overshadowing,” where physical symptoms are mistakenly attributed to their mental illness [8, 9].

Given these risks, physical health checks with a healthcare provider are considered crucial for individuals with SMI, as a way to proactively assess their physical well-being, risk of physical illness, and to offer timely interventions [10]. In the United Kingdom (UK), policy mandates annual physical health checks for adults with SMI [11], which are associated with reduced preventable Accidents and Emergency (A&E) attendances and hospital admissions [12], reducing weight gain, and smoking cessation [5]. These checks typically include assessments of alcohol consumption, blood glucose, blood pressure, body mass index (BMI), lipid profile, and smoking status [8]. Physical health checks aim to detect early signs of conditions like diabetes, stroke, and heart problems, enabling prompt action and earlier intervention. Capturing physical health markers (i.e. BMI, cholesterol, blood glucose) can also determine personal risk profiles for CVD, prompting intervention [13]. Despite these outlined benefits, uptake to these checks remains below the UK target of 73% [14] (58.5% across the 3rd Quarter of 2024-25). In addition, despite National Health Service (NHS) England committing to an increase in annual checks for people with SMI in 2016 [15], the mortality gap has yet to be reversed [1].

Systemic barriers contribute to the poor uptake of physical health checks. Evidence demonstrates the ways in which current healthcare systems are not organised, or accessible, for people with SMI to engage with healthcare in the same way as the general population [16]. In addition, experienced stigma, lack of awareness about the checks, negative past experiences with healthcare, and practical issues like transportation affect access to healthcare in this population [7, 17]. Moreover, there are gaps in training and confidence among primary care clinicians in delivering these checks effectively [5, 9] and in follow-up care [7].

With these barriers in mind, it is apparent that adaptations to current healthcare systems are necessary. Particularly, it is necessary to address this health equity gap in health outcomes, with the aim of improving the uptake of physical health checks for people with SMI [17]. However, with the absence of a systematic review with a specific focus on physical health checks, it is unclear what changes may promote effective improvements in physical health markers and subsequently reduce the risk of conditions such as CVD and diabetes. A recent systematic review examining the effectiveness of collaborative care, integrated care, and physical health interventions for reducing all-cause mortality and promoting cardiovascular health in people with SMI showed moderate support of the use of collaborative care models for improving cardiovascular health in the SMI population [18]. Previous systematic reviews have also focused on lifestyle interventions in SMI [19, 20], without focus on the physical markers for risk of CVD and metabolic conditions. It therefore remains unclear how effective interventions for physical health checks are for reducing risk of CVD and diabetes, as assessed by CV and metabolic markers. This review therefore aims to address this gap by exploring the effectiveness of interventions for improving physical health checks exploring these CV and metabolic markers. In addition, there is an absence of knowledge surrounding the factors that support or hinder implementation of these interventions in practice. Gathering knowledge of implementation is necessary to inform future evidence-based interventions of mechanisms for successful adoption, implementation, and maintenance of physical health check interventions [21].

The current review synthesises available literature reporting on the effectiveness of physical health checks interventions delivered in the community and outpatient care on changing CVD and metabolic markers for people with SMI. Outcomes of this review can inform future research, clinical practice, and policy development to ultimately reduce the significant physical health disparities experienced by individuals with SMI.

### Review question(s)

Through a convergent integrated approach, this mixed methods systematic review aimed to address the following research questions:

*How effective are changes to physical health checks on cardiovascular and metabolic health markers for people living with SMI? Additionally*,* what barriers and facilitators contribute to the implementation of these changes?*

## Methods

This review was conducted in line with the Joanna Briggs Institute Mixed-Methods approach to Systematic Reviews [22] and was reported following the Preferred Reporting Items for Systematic Reviews and Meta-Analysis [23] (PRISMA).

### Information sources

Web of Science, CENTRAL, PsychInfo, SCOPUS and Medline were searched between the 5th February and 10th February 2025.

#### Eligibility criteria

The full eligibility criteria (Table 1) included studies that investigated interventions for improving physical health checks (e.g. BMI, blood pressure, cholesterol), collecting cardiovascular and metabolic markers as their primary outcome. In addition, studies reporting barriers and enablers to implementation of an intervention focused on physical health checks were eligible for inclusion. Interventions had to be targeted at adults with SMI in outpatient care settings. Papers were eligible if they reported studies using quantitative, qualitative or mixed methods. The development of inclusion criteria was done in an iterative process where RH and JC developed the criteria alongside article screening. This was done due to the lack of previous reviews on the biological health markers of physical health checks, and anticipation of heterogeneity of study types (as shown in Langkilde et al., 2025 [18]).

**Table 1:** Eligibility Criteria with definitions

	Inclusion Criteria	Definition
Population	Adults (age 18+) with SMI living in the community and accessing outpatient care. Studies of inpatients were not eligible.	Severe mental illness: People living with severe and enduring mental health conditions such as schizophrenia, bipolar disorder, severe depression, schizoaffective disorder, and psychosis characterised by their impact on the ability to engage in functional and occupational activities [1].
Intervention	Interventions aimed at improving the equity of physical health checks for people with SMI.	**Physical health checks**: A clinical appointment conducted in the community (e.g. primary care) that includes assessments of alcohol consumption, blood glucose, blood pressure, BMI, lipid profile, and smoking status [8] **Intervention**: An activity undertaken with the objective of promoting equitable access to community-delivered physical health checks and prevention of disease [8].
Comparator(s)	No comparator specified	
Outcome(s)	**Primary Outcomes**: - Changes in cardiovascular and/or metabolic health markers **Secondary Outcomes**: - Identification of barriers and enablers to intervention success. - Evidence of effective implementation strategies (e.g., scalability, feasibility). - Measures of equitable access and inclusion of underserved populations.	Cardiovascular and metabolic markers: Physical measurements used to access the health of the heart, blood vessels and metabolic processes and include: Blood sugars (Hba1c), blood pressure, triglycerides, cholesterol, waist circumference/ BMI
Study Designs	All quantitative, qualitative and mixed methods designs. Reviews, conference abstracts, research protocols and audits excluded.	
Other Criteria	Peer-reviewed journal articles, preprints available in English.	

### Search strategy

The search strategy (supplementary file A) was developed in collaboration between all authors. Initially, the strategy outlined in Lamontagne-Godwin [24] was adapted to provide preliminary search results. It was, however, missing terms for SMI and thus, search terms relating to SMI from Coventry [25] were added. The updated strategy was then iterated multiple times by RH and JC and preliminary searches performed in Web of Science, CENTRAL, PsychInfo, SCOPUS and Medline. Once these preliminary searches returned a number of eligible papers, titles and abstracts of initially selected papers were used to create a word cloud using term co-occurrence clustering in VOSviewer [26]. This allowed missing keyword visualisation. In a final step four eligible papers from the preliminary searches were used to conduct a final check for missing key words by scrutinising their abstracts through the Word Frequency Analyser tool of the Systematic Review Accelerator toolbox [27]. Any identified missing words were then added to the search strategy.

### Selection process

Search results were exported into Rayyan [28]. Rayyan’s duplicate detection capabilities were used to remove articles deemed at 97% probability of duplication - any articles below a 97% certainty were manually deduplicated by JC; a 95% threshold has been shown to be 97% accurate [29]. As such, a 97% threshold was chosen to get as close to perfect levels of accuracy, whilst still removing the most clear-cut duplicates. The title, abstract and full text screening process was conducted by JC and RH, and final articles deemed eligible for inclusion were shared with IK and JB for confirmation of inclusion.

### Data items and extraction

Data extraction was conducted in two phases. Firstly, to extract descriptive information, including study design, sample information, and participant information (Table 2), OpenAI’s ChatGPT-4 (gpt-4-base; 8192 token context memory) was used. Supplementary Information B contains the prompts used in the “projects” functionality to generate the specific chatbot that could execute the task with accuracy. JC validated 50% of the output, finding no mistakes. All other data (intervention summaries, evidence of effectiveness and deliverers, required staff training and follow-up and control conditions) were extracted independently by JC and RH into a pre-designed spreadsheet. Extracted items were initially piloted amongst two studies to confirm their suitability. Finally, outputs were discussed in order to validate what each researcher had extracted.

### Quality appraisal

Quality appraisal was conducted using the Mixed Methods Appraisal Tool (MMAT) [30]. All included studies were independently assessed by two researchers (JC, RH), with disagreements resolved in discussion.

### Synthesis methods

The Joanna Briggs Institute (JBI) Convergent Segregated approach to Mixed-Methods Systematic Reviews [29] was followed. This approach involves “independent synthesis of quantitative data and qualitative data leading to the generation of quantitative evidence and qualitative evidence which is then integrated together”. First a data extraction tool was developed iteratively between RH and JC to synthesise study outcomes of both study types (qualitative and quantitative) in a tabular format. This was then discussed with JB and IK to check for completeness. Following this, RH and JC synthesised results following JBI’s methodology, by “qualitising” quantitative data. The process of “qualitisation” involves extracting data from quantitative studies and translating or converting it into ‘textual descriptions’ to allow integration with qualitative data. Ultimately, it involves a narrative interpretation of the quantitative results and is described by Stern [31] as less error-prone than attributing numerical values to qualitative data. Once the process of qualitisation was finished, data were integrated and examined together. This was done through the examination of how the two syntheses fit with each other - how one might explain the outcomes seen in the other, or indeed how they might be in contradiction. As this was done, an identification of “links” between the two sets of data might occur. This ultimately allowed for the construction of arguments based on the two data types, leading to what Stern [31] termed a “configured analysis”.

As such, a narrative synthesis of outcomes was pursued, describing commonalities and differences in outcomes of all studies, as well as possible explanations as to what drove outcomes to be different across studies. A traffic-light approach was used to indicate effectiveness, and significance of intervention findings, when using tabular display of findings. This was done to ensure clarity of findings and goes in line with JBI’s recommendation of synthesis. The traffic-light indicator followed a format of: Green boxes indicate positive, significant, results; red boxes indicate negative, significant, results; and yellow boxes indicate non-significant results.

## Results

### Included studies

In total, 13,795 articles were uploaded to Rayyan [28] of which 3,780 duplicates were removed, leaving 10,015 articles for screening. First, JC and RH screened 996 articles (442 of which overlapped, resulting in less than 10 conflicts). This first batch of decisions was used to train the first version of the Rayyan Support Vector Machine classifier. This classifier’s prediction is presented as split into five categories: Most Likely To Exclude (below 20% threshold of inclusion), Likely To Exclude (below 40%), Likely To Include (Above 60%), Most Likely To Include (Above 80%), and No Recommendation (Not enough information to make recommendation) [32]. In this first batch, the classifier identified 1581 articles that were “Most Likely To Exclude”. These articles were deemed to not fit within the inclusion criteria by the researchers and were removed. JC and RH then performed two more rounds of double screening of 1000 articles to further train the classifier (one conflict, resolved through discussion); 2044 articles were removed in round one, following the classifier’s recommendations (see above), 1781 were removed in the second round. Finally, JC and RH screened the remaining 3,678 articles manually. In the end, 106 articles met eligibility for full-text screening.

**Fig. 1:**
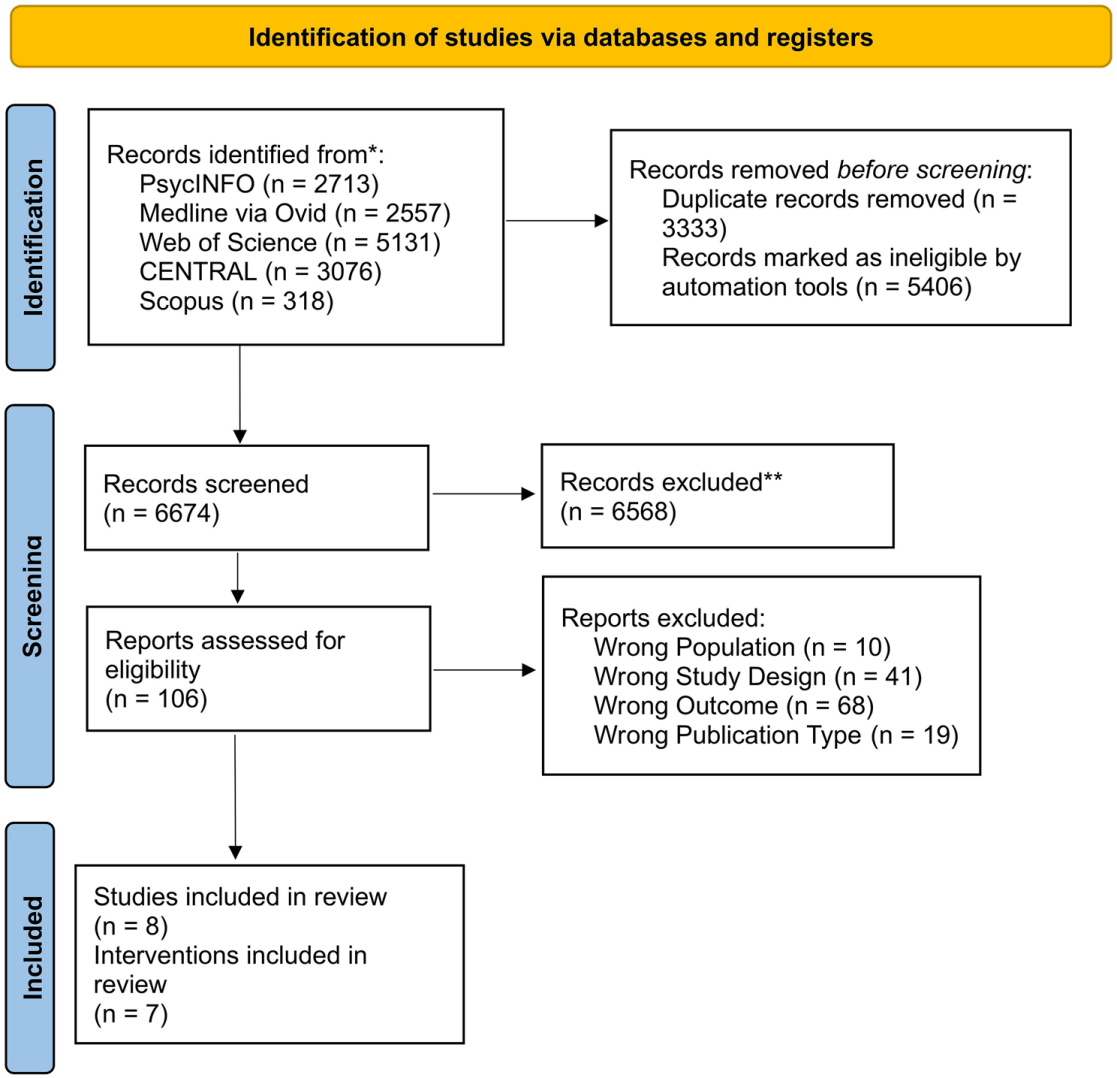
Prisma Flowchart of screening process

JC and RH then screened full text articles independently, with all conflicts resolved in discussion. At this stage, inclusion criteria was tightened to those studies examining physical health check’s effectiveness in biomarker outcome improvement (i.e. excluding studies improving oral checks) in outpatient populations with SMI (i.e. excluding Autistic or disabled populations). Eight papers reporting seven different interventions were included in this review. The study selection process is summarised in Fig. 1.

### Methodological quality

Overall methodological quality of studies, assessed using the 2018 version of the MMAT [30], was varied. Four studies were deemed at low risk of bias (3 RCTs [33, 34, 35] and 1 qualitative study [36]). Whilst the other 4 studies (1 RCT [37] and 3 single-arm studies [38, 39, 40]) showed one area of concern. Three of these four were found to have incomplete reporting, resulting in “Can’t tell” evaluations [38, 40, 41, 36, 38]. Details of the MMAT assessments can be found in Supplementary Material D.

### Characteristics of included studies

Table 2 tabulates study and population characteristics and study outcomes. Of the eight included articles, 7 were quantitative studies: 4 randomised-controlled trials [34, 35, 41], and 3 single-arm studies [38, 40]. One article reported a qualitative study [36]. Most studies were conducted in the United States [35, 38, 41], 2 from the UK [33, 36] and 1 from Denmark [34]. Study sample sizes ranged from 97 to 428 participants in the quantitative designs, and the qualitative study included 30 participants [36]. SMI populations reported by studies were as follows: 5 with Schizophrenia [33, 34, 36, 41], 4 with Bipolar Disorder [33, 35, 41, 36], 2 with Psychosis [33, 36], and 2 with Major Depression Disorder [35, 41]. Other articles did not specify their SMI population [38, 40]. The mean age of participants from across the included articles ranged from 38.8 years to 55.3 years. Four studies included slightly more females (52.4%–63.9%), whereas two were predominantly male [38, 40]. The ethnicity across study samples was varied and included participants who were White, Non-Hispanic White, Black, African American, Asian, and American Indian. One study did not report on ethnicity [34].

**Table 2:** Characteristics of included articles

Study author, date, country	Sample; total sample size	Gender; ethnicity; age of sample	Intervention setting and follow-up	Control condition
**Randomised Controlled Trials**				
Osborn et al. (2018), UK	SMI (schizophrenia, bipolar disorder, psychosis) with CV risk factors; *N* = 327	53% female. 88% White, 5% Black, 3% Asian, 3% Other. Mean age 51 years	Primary care practices 12 months follow-up	Usual primary care with screening feedback but no additional support.
Speyer et al. (2016), Denmark	Schizophrenia; *N* = 428	56.1% female. Ethnicity not reported. Mean age 38.6 years	Community mental health centers 12 months follow-up	Care coordination plus treatment as usual, or treatment as usual alone.
Daumit et al. (2020), USA	SMI (schizophrenia, bipolar disorder, major depression); *N* = 269	52.4% female. 46.1% Black Mean age 48.8 years	Community mental health programs 18 months follow-up	Usual care with exposure to environmental changes (e.g., healthier meal options, physical activity opportunities).
Druss et al. (2010), USA	SMI (schizophrenia, bipolar disorder, major depression); *N* = 407	51.5% male. 77.3% African American Mean age 46.65 years	Urban community mental health 12 month follow-up	Usual care with a list of local medical clinics.
**Single-Arm studies**				
Gonzalvo et al. (2019), USA	Adults with diabetes and on antipsychotics; *N* = 101	59.3% male. 55.6% African American, 42% White Mean age 49.3 years	Primary care clinic within large urban mental health center 2 year follow-up	N/A
Putz et al. (2015), USA	SMI and with metabolic risk factors; *N* = 169	63.9% female 91.1% White, 6.5% Black, 2.4% American Indian Mean age 46.43 years	Community based mental health center 6 month follow-up	N/A
Pirraglia et al. (2012), USA	Veterans with SMI; *N* = 97	95% men 86% non-Hispanic White Mean age 55.3 years	Outpatient mental health clinic 12 month follow-up	N/A
**Qualitative studies**				
Hassan et al. (2020), UK	People with SMI (schizophrenia, bipolar disorder, psychosis); *N* = 15 Healthcare professionals; *N* = 15	People with SMI: 9 male, 6 female, 13 White Staff: 100% female, 100% White British	See Osborn et al. (2018)	N/A

The interventions were conducted in primary care practices [33, 38] and in community mental health centres [33–35, 37, 38, 39, 41]. The one qualitative study [36] included in this review, interviewed people living with SMI (*n* = 15) and staff delivering the intervention (*n* = 15).

#### Intervention content, mode of delivery, staff training and outcomes

The seven interventions to enhance standard physical health checks were diverse in their content, delivery, training protocols and outcomes (outlined in Table 3 and Supplementary Table C). The most common intervention components were individual/lifestyle focused, with a majority adding behaviour change components [33–35, 38, 40, 41] and or dedicated care or case management [34, 35, 39, 41] (5/7 interventions). Other interventions included peer or healthcare professional support [33, 40, 41] (3/7), while a minority implemented structural care changes through establishing new primary care clinics [38, 39].

**Table 3:** Design, Outcome and Effectiveness of Studies

Study author, date, country	Study Design	Summary of Intervention	Study Outcomes	Study Effectiveness
	**Randomised Controlled Trials**			
Osborn et al. (2018), UK	Cluster RCT	Nurse-delivered manualised primary care intervention, including education, support for medication adherence, diet and exercise counseling. Up to 12 sessions	• HDL & LDL*; • PRIMROSE CV risk score; • Systolic & Diastolic Blood pressure; • Lipid concentrations; • HbA1c; • BMI; • Waist circumference;	• Total Cholesterol at 12 months: Mean difference estimate *p* = 0.788; • Secondary Outcomes at 12 months did not differ between the groups • But, resulted in cost-savings and reduced admissions
Speyer et al. (2016), Denmark	Simple RCT	Lifestyle coaching plus care coordination plus treatment as usual, focused on physical activity, diet, and smoking cessation. One-year intervention.	• Copenhagen risk score*; • Waist circumference; • Systolic blood pressure; • Resting heart rate; • Haemoglobin A1c; • HDL and non-HDL cholesterol; • Weight; • Body mass index; • Triglycerides; • High sensitivity C-reactive protein	• Neither group were superior to standard treatment in reducing the Copenhagen risk score (F2,428 = 1.04, *p* = 0.41); • No differences for any secondary outcomes (F2,428 = 0.86, *p* = 0.54);
Daumit et al. (2020), USA	Simple RCT	Behavioral counseling, care coordination, and care management, including weekly sessions with health coaches and nurse visits. 18-month intervention.	• Framingham Risk Score*; • Systolic blood pressure; • Total cholesterol; • High-density lipoprotein; • Diastolic blood pressure; • Fasting blood glucose; • HbA1C; • Low-density lipoprotein; • Triglycerides; • Body mass index	• Framingham Risk Score at 18 months: 12.7% reduction, when compared with control group (95% CI, 2.5%- 22.9%; *P* = 0.02) • Results did not differ by subgroups of age, baseline cardiovascular risk, sex, race, and psychiatric diagnosis. • Between-group differences were not statistically significant for other outcomes.
Druss et al. (2010), USA	Simple RCT	Medical care management intervention with care managers providing advocacy, education, and support	• Framingham Risk Score* (included blood glucose, total cholesterol, and high-density lipoprotein cholesterol)	• Framingham Risk Score significantly lower at 1-year follow-up evaluation (*p* = 0.03) • Intervention group showed an 11.8% rate of improvement (decrease in risk) at the 1-year follow-up evaluation (from 7.8% to 6.9%), and the usual care group showed a 19.5% increase in risk during this period (from 8.2% to 9.8%). This change, while clinically significant, was not statistically significant in the group-by-time interaction.
	**Single-Arm studies**			
Gonzalvo et al. (2019), USA	Retrospective cohort	Pharmacist-managed clinic: medication management, laboratory tests, motivational interviewing, every 6–8 weeks or as needed	• A1C*; • Systolic blood pressure; • Diastolic blood pressure; • non-HDL cholesterol; • Low-density lipoprotein	• Mean A1C decreased by an increment of 0.06% for each month of follow-up time, adjusting for age, gender, and race (*P* < 0.0001). • No significant difference in A1C values between patients on different antipsychotic treatments (*P* = 0.74), controlling for all other covariables. • No significant differences were found between initial and follow-up BP, LDL, and non-HDL values
Pirraglia et al. (2012), USA	Prospective longitudinal	Collaborative care approach with case management, wellness education, physical activity instruction, peer support, and medical care coordination	• Weight/Height; • Systolic blood pressure; • Diastolic blood pressure; • HbA1c; • Total cholesterol; • HDL cholesterol; • Low-density lipoprotein	• Enrollment in SMIPCC was associated with higher goal attainment for blood pressure (adjusted odds ratio [AOR] = 2.16; 95% confidence interval [CI], 1.47–3.18) • Also associated with higher goal attainment for LDL cholesterol (AOR = 1.60; 95% CI, 1.10–2.34), triglyceride (AOR = 1.64; 95% CI, 1.06–2.51), and BMI (AOR = 1.81; 95% CI,1.29–2.54); • No significant difference was found for goal HDL cholesterol or HbA1c. **To understand measurement reporting**: Goal attainment outcomes were based on established performance measures. For example: Goal blood pressure was systolic blood pressure less than 140 mm Hg and diastolic blood pressure less than 90 mm Hg; for patients with diabetes or coronary artery disease, goal blood pressure was less than 130 mm Hg systolic and 80 mm Hg diastolic. See Supplementary Material X for details on other outcomes.
Putz et al. (2015), USA	Retrospective cohort	Co-located, integrated primary care clinic in mental health outpatient clinic, open 1 session/week; open-access scheduling	• Systolic blood pressure; • Diastolic blood pressure; • Body mass index (BMI); • Low-density lipoprotein; • HDL & LDL cholesterol; • Triglycerides; • HbA1c	Results at 6-month follow-up: • BMI: An average 1.47 point (SD D 2.48) reduction in BMI (t(68) = 4.915, *p* < 0.001) • HbA1c: A 0.50% reduction in HbA1c (t(51) = 3.294, *p* = 0.002); • Cholesterol: A nonsignificant decrease of 11.68 mg/dL of total cholesterol: t(15) = 1.425, *p* = 0.175; • HLD: A significant increase in HDL of 2.66 mg/dL: t(37) = 2.58, *p* = 0.016; • LDL: A significant decrease in LDL of 13.64 mg/dL was found: t(24) = 2.169, *p* = 0.040; • Systolic BP: A significant decrease of 15.95 mmHg: t(7) = 4.997, *p* = 0.002; • Diastolic BP: A significant decrease of 8.00 mmHg: t(15) = 3.96, *p* = 0.001;

Note: “*” indicates the study’s primary outcome, lack of asterisk indicates no primary outcome was determined; colour of findings box was determined by the study’s primary outcome, in the case of no primary outcome, this was determined by majority of outcomes; green = significantly positive, yellow = non-significant, red = significantly negative

Modes of intervention delivery varied. Some studies (3/7) used multidisciplinary approaches [34, 38, 40], whilst others [40, 41] relied on single providers. A broad range of professionals delivered the health checks, with nurses being the most common [33–35, 39, 41] (5/7 studies).

Reporting of staff training was inconsistent. Three of seven studies detailed training for delivering interventions [33, 35]. When reported, training was varied in length and content. One study did not specify the content of training [35] but referenced initial and follow up training.

The primary outcomes were also varied, including single markers such as total cholesterol [33], CVD risk score [34, 35], or glycated haemoglobin [38] (HbA1c). Two further studies assessed multiple metabolic markers [39, 40], while another focused on quality of primary care in addition to a CVD risk score [41]. All interventions captured multiple cardiovascular and metabolic markers as secondary outcomes.

### Effectiveness of physical health check interventions

Table 3 displays the effectiveness of each intervention in a “traffic-light” indicator. Overall, 5/7 articles found a significantly positive effect of the intervention on their primary outcome(s) [34–38, 38–41]. The remaining two articles did not [33, 34]. The varied outcomes collected across the interventions are discussed according to their physical health marker category below.

#### Cholesterol

Six studies reported on cholesterol outcomes, with mixed results [33, 35, 38, 40]. A primary care clinic integrated within an outpatient mental health program found significantly higher goal attainment for Low-Density Lipoprotein (LDL) cholesterol (AOR = 1.60; 95% CI, 1.10–2.34) and triglycerides (AOR = 1.64; 95% CI, 1.06–2.51), but not for High-Density Lipoprotein (HDL) cholesterol goals [40]. Whilst another collaborative care intervention found improved LDL and HDL levels, despite no change in total cholesterol [39]. In contrast, four articles evidenced no significant improvements in cholesterol markers [33, 35, 38].

#### Systolic and diastolic blood pressure

Mixed evidence was also found for blood pressure [33, 35, 38, 40] (6/7 studies). Whilst two studies showed significantly positive effects [39, 40], one found modest improvement [35]. The remaining interventions reported no significant improvement in blood pressure [33, 34, 40].

#### Glycated haemoglobin

Six interventions reported on changes to HbA1c, showing similarly mixed results. Two interventions demonstrated significant improvements [38, 39], including people with clinical risk for diabetes (*n* = 52); with an average 0.5% reduction in HbA1c at 6 months shown [39]. However, in one study there was no significant difference in A1C values between patients on different antipsychotic treatments (*p* = 0.74) [38]. The remaining four articles did not evidence a significant effect on glycated haemoglobin [33, 35, 40].

#### Cardiovascular risk scores

Four studies assessed the impact of interventions on overall cardiovascular (CVD) risk scores, yielding conflicting results [33, 34, 41]. Two studies that used the Framingham Cardiovascular Risk Index as a primary outcome found their interventions led to significantly lower 10-year CVD risk score [35, 41]. Conversely, two other trials that used different risk algorithms (including the Copenhagen, QRISK, and PRIMROSE scores) found no significant difference in CVD risk scores between their intervention and control groups [33, 34].

#### Body Mass Index (BMI)

Four of seven interventions captured the impact of their physical health check interventions on BMI [33, 35, 40]. Two studies demonstrated significant success; one reported a significant average reduction in BMI points [39], while the other was associated with higher odds of achieving a goal BMI [40]. However, the two remaining interventions showed no significant effectiveness for improving BMI [33, 34].

### Barriers and facilitators to intervention implementation and success

One qualitative study explicitly explored the barriers and facilitators to implementation of physical health check interventions [36] (outlined in Table 4). In addition, three quantitative studies reflected upon barriers and facilitators to implementation within their discussions [33, 35]. Barriers and facilitators to implementation ranged across five areas: (1) Healthcare knowledge, skills, and attitudes, (2) System integration and workflow compatibility (3) Resource availability and accessibility, (4) Communication and coordination of care and (5) Patient engagement.

**Table 4:** Barriers and facilitators to implementation of physical health check interventions

Themed barrier/facilitator	Barrier to implementation (author, date)	Facilitator to implementation (author, date)
Healthcare provider knowledge, skills, and attitudes	• Stigma and preconceptions of staff towards mental health impacted motivation to deliver the intervention (Hassan et al., 2020) • Lack of knowledge, skills and training especially related to mental health problems (Hassan et al., 2020) • Unclear intervention purpose to people with SMI/healthcare staff (Hassan et al., 2020)	• Appropriate training of healthcare staff (Hassan et al., 2020) • Staff interpersonal skills with people with SMI (Hassan et al., 2020) • Staff motivation to support people with SMI to achieve their health goals (Hassan et al., 2020)
System integration and workflow compatibility	• Busy GP-practices impacted staff willingness to deliver the intervention long-term (Hassan et al., 2020) • Need for additional nurse time to facilitate engagement and accessibility (Hassan et al., 2020) • Variation in team-work practices across different GP practices hindered delivery (Hassan et al., 2020) • Additional workload of specific components (e.g., written health plans) was burdensome and time-consuming (Hassan et al., 2020)	• Positive relationships via supportive team working between healthcare staff in GP practices and staff delivering the intervention (Hassan et al., 2020) • Mandatory examinations of blood lipids in existing health services (Speyer et al., 2016)
Resource availability and accessibility	• Lack of availability of local services for referrals/access to successfully located resources (Hassan et al., 2020; Speyer et al., 2016) • Time to look for referral resources (Hassan et al., 2020)	• Provision of public transportation tokens to ensure patients attended medical visits (Druss et al., 2010) • Help enrolling uninsured patients into programs (Druss et al., 2010) • Existing strategies for troubleshooting factors hindering appointment attendance (Druss et al., 2010)
Communication and coordination of care	• Lack of initiation of statin prescriptions in primary care practices (Osborn et al., 2018)	• Care manager serving as advocate and communication conduit between patient mental health providers (Druss et al., 2010) • Available intervention sheets and explanations from healthcare professionals (Hassan et al.,2020), available information about upcoming appointments (Druss et al., 2010) • Development and maintenance of a provider list by care manager (Druss et al., 2010)
Patient engagement	• Incomplete written health plans ahead of appointments (Osborn et al., 2018) • Complexity of completing health plans (Osborn et al., 2018)	• Use of Motivational interviewing techniques to understand patients’ concerns and reinforce autonomy (Druss et al., 2010) • Action plans and coaching provided to help patients interact more effectively with providers (Druss et al., 2010) • Care manager accompanying patients to visits to specialty providers as needed (Druss et al., 2010)

### Other secondary outcomes

With regards to the additional secondary outcomes of interest in this review (Table 1), we found no evidence across the included articles of effective implementation strategies or measures of equitable access and inclusion of underserved populations adopted by these interventions.

### Integration of quantitative and qualitative evidence

The five interventions conducted in the United States (US) all reported significant positive effect on their various primary outcomes, whilst the two European-led interventions (UK and Denmark) did not (Table 3). These US-based interventions inferred structural differences when compared to the UK and Denmark. This included integrated care models that featured proactive care coordination, the co-location of services within community mental health settings, and the use of multidisciplinary teams that included dedicated care managers, pharmacists, or peer support specialists [38, 41].

Additionally, and highlighting the key role of systemic factors, some of these US-interventions were often part of distinct, funded programs, such as a pharmacist-managed clinic [38] or primary care clinic co-located and integrated in a mental health setting [39]. This implies dedicated resources for the coordination of care. Conversely, the PRIMROSE trial in the UK, which was less successful, attempted to integrate an intervention into existing primary care structures.

The available qualitative evidence infers why an integrated approach is likely a determinant of success. For example, in Druss’ [41] medical care management intervention, facilitators included having a care manager to advocate for people with SMI and provide practical support for resource availability and accessibility barriers.

The qualitative findings from the UK-based PRIMROSE trial [36], which did not demonstrate effectiveness on its primary outcome, highlight the profound challenges of implementing enhanced health checks within standard primary care systems. Additional workload within busy General Practitioner (GP) practices, and a lack of availability of local services for referrals points to systemic issues in supporting intervention implementation. Issues in referral systems were also highlighted in a care coordination [34] intervention, led from Denmark.

An additional noted pattern is that successful interventions were based in, or run out of, community mental health settings [39, 41], whilst the least successful intervention (on primary outcomes) was based entirely in general primary care [33].

## Discussion

### Summary of findings and their implications

This mixed methods systematic review investigated the effectiveness of interventions for physical health checks for improving cardiovascular and metabolic health makers in adults living with SMI, along with the barriers and enablers to their implementation. Eight studies were included (seven interventions) across outpatient (*n* = 2) and community settings (*n* = 5). This relatively small sample highlights the current evidence gap in this area, highlighting a significant need for more research.

While five interventions showed improvements in primary outcomes, results were inconsistent, inferring the need for more consistent approaches. Most interventions combined care coordination with behavioural change techniques (e.g. goal setting, social support), or health behaviour change advice (e.g. physical activity, smoking cessation). Notably, Putz [39] demonstrated significant improvements through an integrated, co-located clinic with open-access scheduling. The use of the open-access scheduling appeared to promote access for this population [41], supporting physical health outcomes. The importance of integrative physical and mental healthcare has been demonstrated previously in SMI research [10, 37, 42]. However, people living with SMI often feel that management for physical health goes excluded due to emphasis on treating the SMI [42].

However, heterogeneity in outcome measures—ranging from CVD risk scores [34, 35, 41] to single markers like A1c [38]—prevented cross-intervention comparison. No single outcome improved across the majority of intervention types, indicating a need for a standardized core outcome set specific to the SMI population. CVD risk scores, for example, are questioned for their generalisability to the SMI population [43].

Furthermore, poor reporting quality and low MMAT scores in 50% of studies limited the ability to identify specific successful components or training requirements. Existing guidance points to the need for consistency and specificity in reporting of intervention development and outcomes [44, 45] This level of detail is important for determining how future implementation of interventions may be impacted [46].

A secondary focus of this review was to determine the barriers and enablers to implementation of these interventions, identifying one qualitative study [36]. Three articles also reflected on implementation in their discussion, whilst 4/7 did not. The absence of implementation consideration possibly contributes to the variability of intervention effectiveness shown in this review. Implementation science reduces variability through systematically understanding and addressing the barriers in practice and addressing inequities in healthcare delivery [47]. Therefore, future research must look to address this evidence gap within the SMI population.

Identified factors influencing success included: healthcare provider knowledge, skills, and attitudes; system integration and workflow compatibility; resource availability and accessibility; communication and coordination of care; and patient engagement. Similar research exploring successful implementation of physical health checks in the SMI population have found that workforce training and supporting navigation of healthcare pathways can support equitable physical healthcare in the SMI population [10]. To seek solutions to these barriers, co-production with key stakeholders (healthcare staff, people with SMI) to determine solutions to overcoming barriers will enable solutions to be collaboratively developed [48].

### Integrated vs. non-integrated care

One seemingly crucial implementation consideration was that of integrated care interventions, also highlighted by similar research in SMI [3]. Interventions that actively restructured delivery to be integrated and coordinated were more successful than those simply adding components to existing services. Integrated models address systemic barriers including diagnostic overshadowing, stigma, and navigation difficulties. For example, in a medication care management intervention [41], the addition of a care manager to support people with SMI interacting with the healthcare system served as an advocate and a communication conduit between patient mental health providers [41].

The interventions included in this review that actively restructured care delivery to be more integrated, coordinated, and proactively managed were more successful. Restructuring of the system may have addressed the systemic barriers such as lack of access to community services and accessible information [5, 7] or “diagnostic overshadowing, [8, 9] that is experienced. Furthermore, current healthcare systems are not organised for people with SMI [16]. And stigma, lack of awareness about the checks, negative past experiences with healthcare, and practical issues like transportation affect access to healthcare in this population [7, 17]. As such, people living with SMI might have benefited from a dedicated team or setting whose role it is to help with the management of the healthcare system itself.

Furthermore, the qualitative results reported by studies in this review found that GP’s had difficulty with referrals, in part due to their busy workload and a lack of availability of local services [34, 36]. Adding to these issues, the gaps in training and confidence among primary care clinicians in delivering these checks effectively [5, 9] might prove to be additional barriers to the type of personalised care people with SMI require. As such, a dedicated team that helps with coordination between different departments and organisational levels might be helpful for the healthcare providers themselves. The specialist skillset and understanding of the SMI population might make these teams, clinics or integrated service providers better equipped to manage both the mental and physical health needs in a coordinated way.

With all of these considerations in mind, future interventions should prioritise improving access equity and removing barriers before attempting to increase the complexity or quality of checks, such as screening for blood-borne viruses and liver function or CV risk assessment [8, 10]. This approach has support in the literature. A recent systematic review examining the effectiveness of collaborative care, integrated care, and physical health interventions for reducing all-cause mortality and promoting cardiovascular health in people with SMI showed moderate support of the use of collaborative care models for improving cardiovascular health in the SMI population [18].

In addition to approaching interventions through the integrative, co-located, lens, future research developing new SMI interventions for physical health checks should also embed experience-based co-production in the design and execution of these interventions. This approach could help to determine what physical health markers are meaningful to people living with SMI [49] and are also recommended in commissioner reports for implementing lifestyle interventions for people with SMI [4] However, embedding co-production within the development of physical health checks interventions appears limited. Therefore, future research is necessary to meaningfully embed service users with lived experience to determine what measures are more meaningful and how co-located clinics and integrated multidisciplinary teams should be added to current healthcare systems, in order to ensure positive outcomes for people living with SMI. In order to support this, funders must look to prioritise funding optimisation trails and research that embeds experience-based co-design in this area of much unmet need.

### Strengths and limitations

This systematic review is the first to synthesise the evidence of effectiveness of physical health checks interventions for improving cardiovascular and metabolic health for people with SMI. This narrow focus is a notable strength. However, due to the limited number of included articles and variability of their methodological approaches and reporting, the inferences made by this review carry limitations. We acknowledge that the comparisons and integrations of findings from across the small sample of heterogeneous interventions should be treated with caution. First, the Primrose intervention is overrepresented in outcomes, due to two included articles reporting this intervention. Specifically, the barriers and facilitators to implementation are more weighted by these results, as the Primrose qualitative study was the only study included in this review that focused specifically on these outcomes. Furthermore, the review was unable to address its secondary outcomes of understanding implementation strategies or measures of equitable access to physical health checks interventions, due to a lack of research. This represents a research gap for future research in this area. Finally, it is possible that eligible studies were not included due to not being indexed in the databases used or were not available in English.

## Conclusion

Significant evidence gaps remain regarding physical health checks for SMI. Despite promising results, limited high-quality studies and inconsistent reporting hinder definitive conclusions. Key priorities for future research include: (1) developing standardised, meaningful outcome measures; (2) improving reporting quality; (3) embedding service-user co-design; (4) using robust methodologies; (5) investigating implementation barriers; and (6) prioritising access equity over the complexity of checks. Funding for optimisation trials and implementation research is crucial to developing sustainable interventions and reducing physical health inequalities in this population.

## Supplementary Information

Below is the link to the electronic supplementary material.

Supplementary Material 1

## Data Availability

No datasets were generated or analysed during the current study.

## Abbreviations

SMI: Severe Mental Illness
MMAT: Mixed Methods Appraisal Tool
CV: Cardiovascular
CVD: Cardiovascular Disease
BMI: Body Mass Index
NHS: National Health Service
A&E: Accidents & Emergency
PRISMA: Preferred Reporting Items for Systematic Reviews and Meta-Analysis
JBI: Joanna Briggs Institute
HbA1c: Glycated Haemoglobin
LDL: Cholesterol Low-Density Lipoprotein Cholesterol
HDL: Cholesterol High-Density Lipoprotein Cholesterol
US: United States
UK: United Kingdom
GP: General Practitioner
