# Supplementary material for: Exploring the effectiveness of physical health check interventions for people with severe mental illness: a systematic review of qualitative and quantitative evidence
Source: BMC Health Serv Res. 2026 Mar 21;26:612. doi: 10.1186/s12913-026-14305-8 (PMC13130709; doi:10.1186/s12913-026-14305-8)
Supplement: Supplementary file 1 — Supplementary Material 1 [file 12913_2026_14305_MOESM1_ESM.docx]

**Supplementary Material**

# Supplementary A: Search strategy (PubMed example)

| PubMed |  |
| --- | --- |
| 1. | (cardiovascular OR vascular OR CVD OR ‘chronic heart disease’ OR ‘coronary heart disease’ OR CHD OR diabetes OR metabolic OR aneurysm) OR cancer OR neoplasm OR carcinoma OR maligna* OR tumour OR tumor OR breast OR mammogra* OR bowel OR cervical OR pap OR (dental OR dentist OR tooth OR teeth) OR (eye OR retinopathy) OR (inequal* or disparit* or inequit*) OR ((breast* or bowel* or colorect* or lung* or cervic*) adj3 (cancer* or neoplasm* or tumour* or tumor* or malignan* or carcino*)) |
|  | AND |
| 2. | (‘mass screening’ OR surveillance*) OR “Screening Test” OR ((cholesterol OR fecal OR faecal OR blood OR HIV OR sigmoid OR tuberculosis) AND test*) OR “health check*” OR monitoring OR screen* OR smear* OR mammogra* OR test* OR exam* OR detect* OR (primary care OR primary health care OR primary healthcare) OR (general practi* OR family practi* OR family medicine OR GP) OR ("Physicians, Family") OR exp primary health care/ OR exp primary medical care/ OR exp general practice/ OR exp general practitioner/ |
|  | AND |
| 3. | Exp intervention/ OR exp diffusion of innovation/ OR exp Technology Transfer/ OR exp Translational Medical Research/ OR exp Organizational Innovation/ OR exp Health Plan Implementation/ OR Information Dissemination/ OR implement* OR dissemin* OR (adoption or adopter or adopters or adaptation or adapt) OR (organi?ational innovation* OR organi?ational change*) OR (diffuse or diffusion) OR ((system or systems or systematic or systematically) adj2 (change or changer or changed or changes)) OR organi?ational innovation* OR sustainab* OR institutionali* OR routin* OR maintenance OR incorporat* OR integrat* OR (Implement* adj3 fidelity) OR quality improvement* OR Facilitat* OR Barrier* OR ((knowledge OR technolog* OR research) adj3 (transform* OR translat* OR exchange or transfer OR integration OR utilization)) OR Training/ OR Training OR Education |
|  | AND |
| 4. | satisf* OR dropout* OR ‘drop out’ OR attrition OR uptak* OR adher* OR compliance OR complie* OR comply* OR ‘patient acceptance of health care’ OR encourag* OR improve* OR improving OR increas* OR promot* OR particip* OR nonattend* OR ‘non attend’ OR accept* OR attend* OR attitud* OR utilisation OR utilization OR refus* OR respond* OR respons* OR reluctan* OR nonrespon* OR ‘non respon*’ OR incidence OR prevalence OR prevalence OR satisfaction OR cooperat* OR ‘co operat*’ OR service* OR support* OR engagement |
|  | AND |
| 5. | (bipolar adj (disorder* or disease* or illness*)) OR exp schizophrenia/ OR Affective disorders, psychotic/ OR Bipolar disorder/ OR paranoid disorders/ OR exp psychotic disorders/ OR schizo* OR (mani* adj3 depress*) OR (psychotic* adj3 depress*) OR (severe* adj3 affective*) OR (severe* adj3 mental*) OR (severe* adj3 depress*) OR (psychos?s adj3 depress*) OR (serious* adj3 affective*) OR “serious mood*” OR (serious* adj3 depress*) OR (persons with mental disabilities/ OR mentally ill persons/ OR intellectual disability/) OR (autism OR autistic* OR Exp autistic disorder/ OR Exp autism spectrum disorder OR Exp Asperger syndrome/ OR psychiatric disorder* OR mental health illness*) |

#

# Supplementary Information B - Chatbot Prompts:

## *ChatGPT Project File Prompts:*

### *Randomised Controlled Trials*

“Extract the following from the PDF - accuracy is vital - never speculate and be clear if you

don't know. You are assessing quantitative studies with a control group

1) Very short description of the sample (This should include eligibility criteria like type of

diabetes, comorbidities, diabetes management or treatment, or other specific criteria like

ethnicity or deprivation)

2) Total sample size in terms of number of people - this is important - it should equal the total

of all conditions. If there are just two conditions, sum the n in these to get the total n.

3) % split between men and women

4) % split by ethnicity

5) Age range and mean

6 ) Very short description of the study setting

7) The longest duration from the intervention to the follow-up data collection

8) Very short description of the intervention (Keep this concise. What was done to people

and for how long. Did they only get CGM or something else as well?) and N of the

intervention group

9) Very short description of the comparator or control condition (Keep this concise. What was

done to people and for how long) and N of the comparator group

10) Very short Description of the study design

11) Primary study outcome and specific measure used

Format that in a row where there are 6 columns and 3 rows, with the column 1 containing

items 1 and 2, column 2 containing items 3, 4 and 5, column 3 containing items 6 and 7,

column 4 containing item 8, column 5 containing column 9, and column 6 containing items

10 and 11. Items should be on their own row in the column, with a line return separating the,

from the previous item, making the new item clearly separate”

### *Qualitative Studies*

“Extract the following from the PDF - accuracy is vital - never speculate and be clear if you

don't know.

1) Short description of the sample (This should include eligibility criteria like type of diabetes,

comorbidities, diabetes management or treatment, or other specific criteria like ethnicity or

deprivation)

2) Total sample size in terms of number of people.

3) % split between men and women

4) % split by ethnicity

5) Age range and mean

6 )Short description of the study setting

7) short Description of the context of the qualitative data collection (Was this a standalone

qual study or part of a trial, eg process evaluation?)

8) short description of the intervention (Keep this concise. What was done to people and for

how long. Did they only get CGM or something else as well?)

Format that in a row where there are 4 columns and 3 rows, with the column 1 containing

items 1 and 2, column 2 containing items 3, 4 and 5, column 3 containing items 6 and 7, and

column 4 containing item 8. Items should be on their own row in the column, with a line

return separating the, from the previous item, making the new item clearly separate”

### *Single Group Studies*

“1) Very short description of the sample (This should include eligibility criteria like type of diabetes, comorbidities, diabetes management or treatment, or other specific criteria like ethnicity or deprivation)

2) Total sample size in terms of number of people.

3) % split between men and women

4) % split by ethnicity

5) Age range and mean

6)very Short description of the study setting

7) The longest duration from the intervention to the follow-up data collection

8) very short description of the intervention (Keep this concise. What was done to people and for how long. Did they only get CGM or something else as well?) and N of the intervention group

9) very short Description of the study design

10) Primary study outcome and specific measure used Format that in a row where there are 5 columns and 3 rows, with the column 1 containing items 1 and 2, column 2 containing items 3, 4 and 5, column 3 containing items 6 and 7, column 4 containing item 8, and column 5 containing items 9 and 10. Items should be on their own row in the column, with a line return separating the, from the previous item, making the new item clearly separate”

# Supplementary C: Data synthesis table

| **Author; year** | **Intervention delivered by** | **Staff training** | **Evidence of effectiveness** | **Barriers to implementation** | **Facilitators to implementation** | **Implementation strategies** | **Measures of equitable access** |
| --- | --- | --- | --- | --- | --- | --- | --- |
| Osborn et al (2018) | Nurses and healthcare assistants | Two day training package and manual. Staff trained on two occasions to use the behaviour change strategies. | Total cholesterol between intervention and control group did not differ at 12 months.  Mean total cholesterol decreased in both groups over the 12-month follow-up period.  Secondary clinical outcomes did not differ between the groups at 12 months. | - Lack of initiation of statin prescriptions in primary care practices  (see Hassan et al., 2020) | See Hassan et al. (2020) | Does not report | Does not report |
| Hassan et al., 2020 | As above (Osborn et al., 2018) | As above (Osborn et al., 2018) | As above (Osborn et al., 2018) | - Unclear intervention purpose to people with SMI/healthcare staff.  -Stigma and preconceptions of staff towards mental health impacted motivation  - Contextual integration within busy GP-practices if implemented in the future, impacts staff willingness to deliver long-term.  -Additional workload of specific components of the intervention  - Additional nurse time to facilitate engagement and accessibility to the intervention.  -Variation in teamwork practices across different GP practices  -Limited time during appointments to complete written health plans  -Uncompleted written health plans  - Lack of knowledge, skills and training related to mental health (staff)  - Lack of availability of local services for referrals | -Clarity of the intervention purpose  -Appropriate intervention sheets and explanations from healthcare professionals.  -High staff motivation  - Positive relationships between healthcare staff in the GP practices and the staff delivering the intervention  - Appropriate training of healthcare staff  -Staff interpersonal skills with people with SMI | Does not report | Does not report |
| Putz et al., 2015 | Social worker, family nurse-practitioner, 2 nurse managers,peer-support specialist/certiﬁed recovery specialist, wellness coach, ofﬁce professional. | Does not report | (n=63) (67.74%) lost weight at 6-m follow up. At 6-m follow up, average reduction in weight from baseline was 2.80%.  (n=52) At 6-m on average, a 0.50% reduction in HbA1c,  (n=16) A non sig. decrease of 11.68 mg/dL of total cholesterol at 6-m found.  A statistically sig. increase in HDL of 2.66 mg/dL at the six-month follow-up. A statically signiﬁcant decrease in LDL of 13.64 mg/dL at the six-month follow-up  Systolic BP (n=8) A statistically sig. decrease of 15.95 mmHg at the six-month follow-up:  Diastolic BP (n=16) A sig. decrease of 8.00 mmHg in diastolic blood pressure at six month | Does not report | Does not report | Does not report | Does not report |
| Speyer et al., 2016 | Lifestyle coaches, care-coordinator (psychiatric nurse), GP | 5-day courses on MI and smoking cessation, 1-day course in examination and treatment of lifestyle disorders, and a 2-day course in healthy dieting. | Neither the CHANGE intervention or care coordination were superior to standard treatment for reducing 10-year CVD risk.  The mean age-standardized 10-year risk of CVD was 8.4 ± 6.7% in the CHANGE group, 8.5 ± 7.5% in the care coordination group, and 8.0 ± 6.5% in control group.  No differences between the three groups for any of the secondary outcomes.  No sig. differences between the three groups on exploratory outcomes. | -Lack of referrals | -Mandatory examinations of blood lipids in existing health services | Does not report | Does not report |
| Gonzalvo et al (2019) | Pharmacist | Does not report | A sig. mean A1C decreased by an increment of 0.06% for each month of follow-up time, adjusting for age, gender, and race.  No sig. differences found between initial and follow-up BP, LDL, and non-HDL values | Does not report | Does not report | Does not report | Does not report |
| Druss et al (2010) | Nurses | Does not report | Framingham CV Risk Index score was sig. lower at the 1-year follow-up in the intervention group vs usual care group (6.9% versus 9.8%)  Intervention group showed an 11.8% rate of improvement (decrease in risk) at the 1-year follow-up evaluation (from 7.8% to 6.9%), and the usual care group showed a 19.5% increase in risk during this period (from 8.2% to 9.8%). | Does not report | - Enhancing motivation by providing information to patients.  -Use of information booklets  -Motivational interviewing techniques helped support patients’ self-management skills  -Action plans were used to foster health behavior change  - Advocating for the patient  - Supporting joined up communication between the patient and specialty medical and mental health providers.  -Coaching patients to help them interact more effectively with their providers.  - Public transportation tokens to ensure that patients were able to attend all medical visits. | Does not report | Does not report |
| Daumit et al. (2020) | Health coaches, nurse | Initial and follow-up training and regular observations. | Net reduction in the 10-year global Framingham Risk Score for the intervention group vs control at 18 months was 12.7%. Analyses using American College of Cardiology and American Heart Association Risk Score showed net percentage reduction in 10-year CVr risk of 13.2% for the intervention vs control at 18 months.  Each risk score component measured in continuous scale (mean [SD)] at 18 months in the intervention vs control group was lower. But, between-group differences were not statistically sig.  For binary risk score components, absolute changes in prevalence for diabetes and medication for hypertension also were both nonsig.for intervention vs control at 18 months. | Does not report | Does not report | Does not report | Does not report |
| Pirraglia et al (2012) | A single primary care provider and a patient care assistant | Does not report | Enrollment in the integrated primary care clinic was associated with higher goal attainment for blood pressure, LDL cholesterol, triglyceride and BMI. No sig. difference was found for goal HDL cholesterol or HbA1c. | Does not report | Does not report | Does not report | Does not report |

#

# Supplementary Information D: Mixed Methods Appraisal Tool and Outcomes of Quality Appraisal

## *Mixed Methods Appraisal Tool 2018 Version.*

| **Category of study designs** | **Methodological quality criteria** | **Responses** | | | |
| --- | --- | --- | --- | --- | --- |
|  |  | **Yes** | **No** | **Can't tell** | **Comments** |
| **Screening questions**  **(for all types)** | S1. Are there clear research questions? |  |  |  |  |
|  | S2. Do the collected data allow to address the research questions? |  |  |  |  |
| **1. Qualitative** | 1.1. Is the qualitative approach appropriate to answer the research question? |  |  |  |  |
|  | 1.2. Are the qualitative data collection methods adequate to address the research question? |  |  |  |  |
|  | 1.3. Are the findings adequately derived from the data? |  |  |  |  |
|  | 1.4. Is the interpretation of results sufficiently substantiated by data? |  |  |  |  |
|  | 1.5. Is there coherence between qualitative data sources, collection, analysis and interpretation? |  |  |  |  |
| **2. Quantitative**  **randomized controlled**  **trials** | 2.1. Is randomization appropriately performed? |  |  |  |  |
|  | 2.2. Are the groups comparable at baseline? |  |  |  |  |
|  | 2.3. Are there complete outcome data? |  |  |  |  |
|  | 2.4. Are outcome assessors blinded to the intervention provided? |  |  |  |  |
|  | 2.5 Did the participants adhere to the assigned intervention? |  |  |  |  |
| **3. Quantitative nonrandomized** | 3.1. Are the participants representative of the target population? |  |  |  |  |
|  | 3.2. Are measurements appropriate regarding both the outcome and intervention (or exposure)? |  |  |  |  |
|  | 3.3. Are there complete outcome data? |  |  |  |  |
|  | 3.4. Are the confounders accounted for in the design and analysis? |  |  |  |  |
|  | 3.5. During the study period, is the intervention administered (or exposure occurred) as intended? |  |  |  |  |
| 4. Quantitative  descriptive | 4.1. Is the sampling strategy relevant to address the research question? |  |  |  |  |
|  | 4.2. Is the sample representative of the target population? |  |  |  |  |
|  | 4.3. Are the measurements appropriate? |  |  |  |  |
|  | 4.4. Is the risk of nonresponse bias low? |  |  |  |  |
|  | 4.5. Is the statistical analysis appropriate to answer the research question? |  |  |  |  |
| **5. Mixed methods** | 5.1. Is there an adequate rationale for using a mixed methods design to address the research question? |  |  |  |  |
|  | 5.2. Are the different components of the study effectively integrated to answer the research question? |  |  |  |  |
|  | 5.3. Are the outputs of the integration of qualitative and quantitative components adequately interpreted? |  |  |  |  |
|  | 5.4. Are divergences and inconsistencies between quantitative and qualitative results adequately addressed? |  |  |  |  |
|  | 5.5. Do the different components of the study adhere to the quality criteria of each tradition of the methods involved? |  |  |  |  |

##

## *Current Review’s Quality Appraisal Outcomes*

| Randomised Controlled Trials (RCT) | | | | | | | |
| --- | --- | --- | --- | --- | --- | --- | --- |
| MMAT Question | S1 | S2 | 2.1 | 2.2 | 2.3 | 2.4 | 2.5 |
| Druss et al. (2010) | No | Yes | Yes | Yes | Yes | Can’t Tell | Can’t Tell |
| Speyer et al. (2016) | Yes | Yes | Yes | Yes | Yes | Yes | Yes |
| Daumit et al. (2020) | Yes | Yes | Yes | Yes | Yes | Yes | Yes |
| Osborn et al. (2018) | Yes | Yes | Yes | Yes | Yes | Yes | Yes |
| Quantitative Non-Randomised | | | | | | | |
| MMAT Question | S1 | S2 | 3.1 | 3.2 | 3.3 | 3.4 | 3.5 |
| Putz et al. (2015) | Yes | Yes | Yes | Yes | Yes | No | Yes |
| Pirraglia et al (2012) | Yes | Yes | Can’t Tell | Yes | No | Yes | Yes |
| Gonzalvo et al (2019) | Yes | Yes | Can’t Tell | Yes | No | Can’t Tell | No |
| Qualitative | | | | | | | |
| MMAT Question | S1 | S2 | 1.1 | 1.2 | 1.3 | 1.4 | 1.5 |
| Hassan et al. (2020) | Yes | Yes | Yes | Yes | Yes | Yes | Yes |
